# An Unusual Case of Neurofascin 186 Neuropathy

**DOI:** 10.7759/cureus.63049

**Published:** 2024-06-24

**Authors:** Khushboo S Hatekar, Shalesh Rohatgi, Satish P Nirhale, Prajwal M Rao, Pravin U Naphade

**Affiliations:** 1 Department of Neurology, Dr. D. Y. Patil Medical College, Hospital & Research Centre, Dr. D. Y. Patil Vidyapeeth, Pune, IND

**Keywords:** immune-mediated neuropathy, rituximab therapy, therapeutic plasmapheresis, guillian barré syndrome, chronic inflammatory demyelinating polyneuropathy, inflammatory neuropathy

## Abstract

Among the diverse array of neuropathies, autoimmune neuropathy stands out as a distinctive subset, where the body's immune system mistakenly attacks its nerve tissues, triggering inflammation and nerve damage. NF 186, also known as neurofascin 186, is a cell adhesion molecule crucial for the integrity and functioning of the peripheral nervous system. This case report highlights the clinical presentation specific to NF 186-positive autoimmune neuropathy and also the treatment modalities.

## Introduction

Nodo-paranodopathy was first introduced to describe neuropathies with antiganglioside antibodies which share a common underlying dysfunction at the node. This dysfunction leads to a continuum of pathophysiological effects ranging from reversible nerve conduction abnormalities to complete axonal degeneration. This concept was first described in an axonal variant of Guillain-Barré syndrome (GBS) with positive anti-ganglioside antibodies. The spectrum expanded later to include demyelinating neuropathies such as chronic inflammatory demyelinating polyradiculoneuropathy (CIDP) and other neuropathies secondary to immune, inflammatory, ischemic, nutritional, and toxic aetiologies [1].

Patients with neurofascin 186 (NF186)-positive autoimmune neuropathy exhibit acute or subacute onset disease courses, often resembling GBS or presenting as acute attacks within a chronic course. Weakness in NF186-positive patients is not confined to distal extremities but also affects proximal limbs, resembling features seen in classic CIDP. Additionally, asymmetrical involvement is frequent. Despite the presence of sensory disturbances and ataxia, these symptoms are less frequent in NF186-positive patients.

Patients with antibodies that cross-react with both neurofascin isoforms (NF155 and NF186) may experience a rapid onset of neuropathy. The involvement of the cranial nerves and the presence of autonomic dysfunction and respiratory failure are common.

## Case presentation

A 50-year-old male presented with insidious onset distal symmetric weakness and tingling sensation in bilateral hands for one year. The weakness gradually progressed to involve the proximal upper limbs over the year. He noticed that his hands began to become thinner. He developed symmetric proximal and distal bilateral lower limb weakness, which progressed gradually over six months. He also complained of tingling and numbness in bilateral feet up to the ankle for the last six months.

He had a fever for two days followed by breathlessness and was admitted to an outside hospital in February 2024. He was intubated for respiratory failure and was then referred to our hospital for further management. There was no history of swallowing difficulty, chronic cough, visual complaints, urinary or bowel complaints, muscle twitching, tremulousness of hands, headache, recent vaccination, medication/toxin exposure, loss of weight, or trauma.

On examination, he was conscious and obeying commands. His pulse was 100 beats per minute. He had normal blood pressure. He was maintaining a 60% fraction of inspired oxygen (FiO2) on ventilatory support in volume control mode. His neurological examination revealed hypotonia in bilateral upper limbs and lower limbs. Bilateral upper limb muscle strength of grade 3 was present at the shoulder and elbow joint and grade 2 at the wrist. Muscle strength in lower limbs was grade 3 at the hip and knee joint and grade 2 at the ankle bilaterally. Muscle atrophy was seen in bilateral upper limbs and lower limbs with severe wasting of small muscles of hands and feet. Areflexia was present in all four limbs. His planters were flexor. Peripheral nerves were not thickened. Sensory examination could not be performed as the patient was intubated. Other neurological examinations were normal.

Laboratory tests showed a normal haemogram. Liver function tests, renal function tests, electrolytes, hepatitis B surface antigen, serum human immunodeficiency virus antibody, antinuclear antibody test, vitamin B12 levels, creatinine phosphokinase, thyroid profile, syphilis rapid plasma reagin, and serum protein electrophoresis were normal. Serum immunofixation for immunoglobulin analysis, acetylcholine receptor antibodies, and anti-muscle-specific kinase (anti-MuSK) antibodies were all negative. High-resolution CT thorax showed ill-defined areas of consolidation involving the right lower lobes and left upper lobe, features likely representing infective aetiology.

His brain and spine MRI showed no obvious abnormalities. Cerebrospinal fluid analysis showed a protein of 42 mg/ dl, the leukocyte count was 2 with lymphocytic predominance, and he had a glucose level of 55mg/dl with a corresponding blood sugar level of 106mg/dl.

His nerve conduction study showed decreased compound motor action potentials and decreased conduction velocity in bilateral median and ulnar nerves and left peroneal and tibial nerves and absent sensory nerve action potential in bilateral ulnar, median, and sural nerves with reduced conduction velocity and normal F wave latency (Table 1).

**Table 1:** Nerve conduction study (NCS)

Motor NCS	Latency (ms)	Amplitude (mV)	Conduction velocity (m/s)	F wave latency (ms)
Left median				
Wrist	4.1	2.7	35.8	29
Elbow	10.2	1.1		
Right median				
Wrist	3.4	1.9	30	28
Elbow	10.7	0.8		
Left ulnar				
Wrist	3.1	1.7	48	24
Elbow	8.1	0.4		
Right ulnar				
Wrist	2.6	1.9	41.4	26
Elbow	8.4	0.6		
Right peroneal				
Ankle	5.3	2.9	34.4	51
Head of fibula	13.9	2.6		
Left peroneal				
Ankle	4.3	0.3	42.1	24
Head of fibula	14.8	0.3		
Right tibial				
Ankle	4.3	4.0	28.5	57
Popliteal	17.3	3.0		
Left tibial				
Ankle	4.6	1.8	31.5	53
Popliteal	17.3	1.1		
Sensory NCS				
Bilateral median	Absent	Absent	Absent	Absent
Bilateral ulnar	Absent	Absent	Absent	Absent
Bilateral sural	Absent	Absent	Absent	Absent

**Figure 1:**
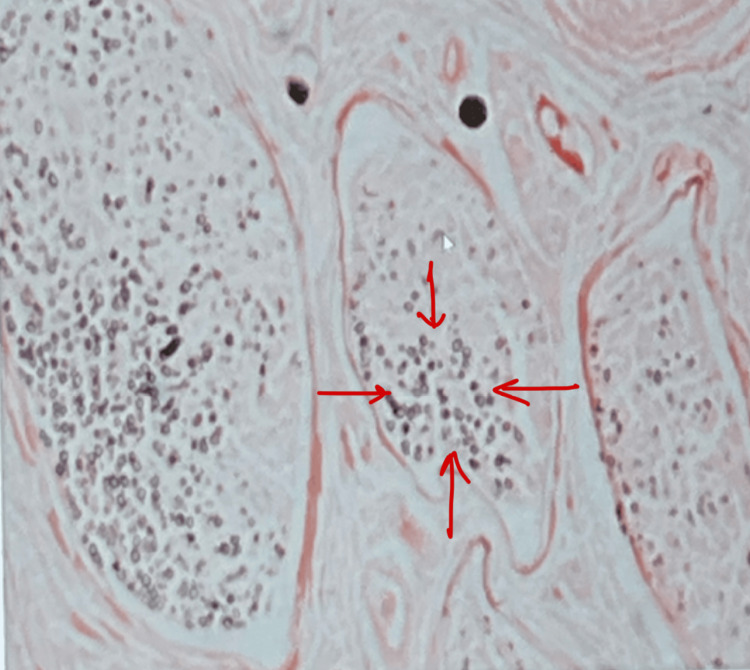
Sural nerve biopsy

A nerve biopsy of the left sural nerve showed chronic mild asymmetric axonal and demyelinating neuropathy features as shown in Figure 1.

The above-given reports and a subacute onset, progressive sensorimotor neuropathy with respiratory failure, led to the suspicion of pan-NF-antibody-associated neuropathy. Serums NF155 and NF186 were sent and reports were awaited.

The patient was initiated on a five-day course of IV methylprednisolone 1 gm/day followed by plasmapheresis. Seven cycles were completed along with IV antibiotics for lower respiratory tract infection, bronchoalveolar lavage was done, and antibiotics were stepped up as per culture sensitivity. However, there was difficulty in weaning off from ventilatory support even after the resolution of pneumonia for 20 days.

Serum NF186 antibodies were positive. IVIg 20 grams/day was given for five days followed by an induction dose of IV 1 gm rituximab infusion as per protocol. The patient had mild improvement in muscle strength, however he required continuous positive airway pressure ventilation.

## Discussion

Neuropathies with nodal and paranodal antibodies are autoimmune nodopathies as per the 2021 European Academy of Neurology/Peripheral Nerve Society (EAN/PNS) criteria and are independent of CIDP. Each CIDP subtype related to specific autoantibodies has distinct clinical features. It is recommended to screen for anti-NF186 antibodies in patients suspected of acquired acute-onset neuropathy to ensure accurate diagnosis and appropriate management [2].

**Figure 2:**
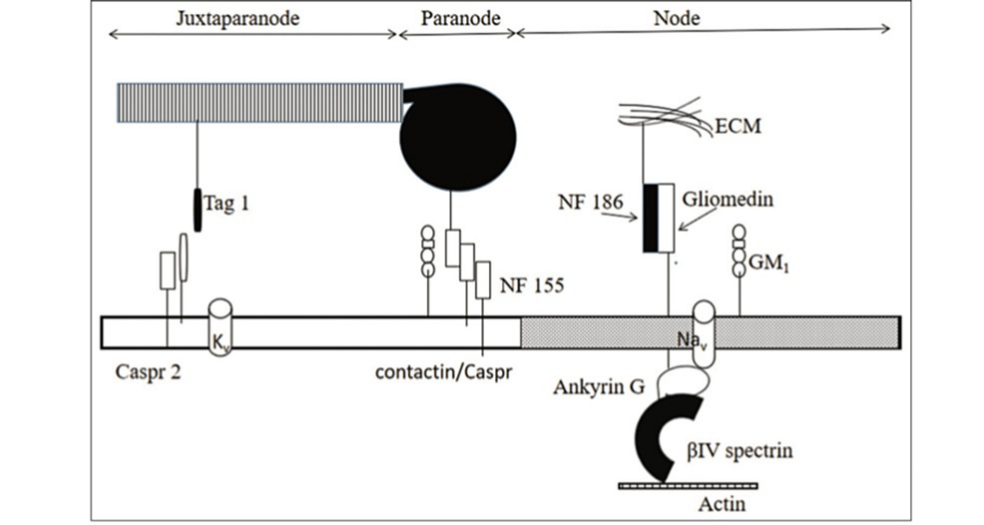
Anatomy and molecular organization of the node Image Credit: Khadilkar et al., 2022 [1]; open access

The anatomy and molecular organization at the node is as shown in Figure 2 [1].

Weakness in NF186-positive patients is not confined to distal extremities but also affects proximal limbs, resembling features seen in classic CIDP. Asymmetrical involvement is common, necessitating differentiation from focal CIDP or Lewis-Sumner syndrome. Despite the presence of sensory disturbances and ataxia, these symptoms are less common in NF186-positive patients [3,4].

Patients with NF155 and NF186 have sudden and rapid progression of weakness to involve all four limbs along with the involvement of cranial nerve respiratory and autonomic dysfunction [5]. Three cases showed membranous nephropathy with a positive presence of anti-pan-NF antibodies, and two cases exhibited a positive presence of anti-NF186 antibodies [6]. While some patients responded positively to IVIg, they had a rapid relapse within a few days or weeks [7]. They required ICU management for a prolonged period. The response to plasmapheresis and corticosteroids was not effective for patients with antibodies to pan-NF, resulting in high mortality rates despite some cases of spontaneous remission. The response to rituximab therapy was good. This occurrence is uncommon compared to anti-contactin 1 (CNTN1) antibody-positive patients and may be due to the presence of NF186 in podocytes of the glomerular basement membrane [6,8].

Nerve conduction studies showed initially slow conduction velocities, which were thought to be caused by demyelination. However, findings with reduced amplitudes or unrecordable compound muscle action potentials (CMAPs) were found at follow-up in five patients [8]. Nerve biopsy showed axonal loss, however, without any signs of inflammatory changes or segmental demyelination in two patients [9]. Patients with anti-CNTN1 antibodies present with an acute or subacute onset course with a prevalent distal weakness, ataxia, and no or poor response to IVIg [10,11]. Anti-CNTN1 antibodies were also reported in patients with an acute presentation [12]. A primary autoimmune membranous nephropathy can be associated with anti-CNTN1-related neuropathy [13]. Anti-Caspr1 antibodies were first seen in a patient with an acute inflammatory demyelinating polyneuropathy (AIDP)-like presentation and also with a subacute or chronic course similar to chronic inflammatory demyelinating neuropathy with exacerbations during infections. Response to IVIg was poor [14]. Neuropathic pain was present in patients. There were 15 patients showing immunoreactivity against the Caspr1 [15]. In patients meeting the EFNS/PNS criteria for definite CIDP, the frequency of antibodies to Caspr1 and CNTN1 complex was 1.9% in a Barcelona, Spain, cohort and 4.3% in a German cohort of acute-onset CIDP. Almost half of the patients were initially diagnosed with GBS due to acute onset, and a high percentage of them experienced ataxia, tremors, the involvement of cranial nerves, and the presence of neuropathic pain. Standard treatments had limited effectiveness, but rituximab showed a 90% response rate, although recovery was generally slow for most patients [16].

## Conclusions

Presenting with insidious onset and gradually progressive sensorimotor polyneuropathy accompanied by respiratory failure, our patient's normal cerebrospinal fluid examination and axonal sensory-motor neuropathic presentation on nerve conduction study further supported the diagnosis of NF186-positive autoimmune neuropathy. Prompt initiation of plasmapheresis followed by IV steroids and rituximab therapy resulted in a favourable response, underscoring the importance of timely and targeted interventions in managing this complex condition.
